# Complexes With Biologically Active Ligands. Part 6^1^ Ni(II) Coordination Compounds of Hydrazine and Heterocyclic Sulfonamides as Inhibitors of the Zinc Enzyme Carbonic Anhydrase

**DOI:** 10.1155/MBD.1996.143

**Published:** 1996

**Authors:** Joaquin Borràs, Javier Casanova, Teodora Cristea, Adriana Gheorghe, Andrea Scozzafava, Claudiu T. Supuran, Violeta Tudor

**Affiliations:** 1 University of Valencia Faculty of Pharmacy Department of Inorganic Chemistry, Vincent Andres Estelles s/n Burjassot (Valencia) E-46100 Spain; 2 University of Bucharest Department of Chemistry Bd. Carol I (Republicii) 13 Bucharest R-70346 Romania; 3 University of Florence Laboratory of Inorganic and Bioinorganic Chemistry, Via Gino Capponi 7 Firenze I-50121 Italy

## Abstract

Ternary Ni(II) complexes of hydrazine and eight heterocyclic sulfonamides possessing carbonic anhydrase (CA) inhibitory properties, were prepared and characterized by elemental analysis, spectroscopic, magnetic, thermogravimetric, and conductimetric measurements. The complexes behave as strong inhibitors for two isozymes (I and II) of carbonic anhydrase.


### COMPLEXES WITH BIOLOGICALLY ACTIVE LIGANDS. Part 6 [1] Ni(II) COORDINATION COMPOUNDS OF HYDRAZINE AND HETEROCYCLIC SULFONAMIDES AS INHIBITORS OF THE ZINC ENZYME CARBONIC ANHYDRASE[#]

Joaquin Borràs[1], Javier Casanova[1], Teodora Cristea[2], Adriana Gheorghe[2], Andrea Scozzafava[3], Claudiu T. Supuran*[3] and Violeta Tudor[2]

[1] University of Valencia, Faculty of Pharmacy, Department of Inorganic Chemistry, Vincent Andres Estelles s/n, E-46100, Burjassot (Valencia), Spain
[2] University of Bucharest, Department of Chemistry, Bd. Carol I (Republicii) 13, R-70346 Bucharest, Roumania
[3] University of Florence, Laboratory of Inorganic and Bioinorganic Chemistry, Via Gino Capponi 7, I-50121, Firenze, Italy

**Abstract**: Ternary Ni(II) complexes of hydrazine and eight heterocyclic sulfonamides possessing carbonic anhydrase (CA) inhibitory properties, were prepared and characterized by elemental analysis, spectroscopic, magnetic, thermogravimetric, and conductimetric measurements. The complexes behave as strong inhibitors for two isozymes (I and II) of carbonic anhydrase.

### Introduction

Heterocyclic sulfonamides constitute a class of strong inhibitors of the zinc enzyme carbonic anhydrase (CA, EC 4.2.1.1), and have wide clinical applications in the treatment of a variety of disorders such as glaucoma, gastro-duodenal ulcers, and acid-base disequilibria among others.[2] Compounds **1-6** are all clinically used such drugs, with acetazolamide **1**, methazolamide **2** and ethoxzolamide **3** being first generation inhibitors (without specificity for different CA isozymes or selectivity for diverse organs where these enzymes are present), benzolamide **5** and chlorzolamide **6** having the status of orphan [3] or experimental drugs[4] (and also showing specificity for some CA isozymes in certain concentrations[3,4]), whereas sezolamide **4** is the representative of a novel class of such inhibitors (third generation) recently introduced in clinical medicine as topically effective drugs in the treatment of glaucoma.[5] 5-Amino-1,3,4-thiadiazole-2-sulfonamide **7** is on the other hand the key compound for synthesis of diverse inhibitors such as **1**, **2**, **5** or **8**. This last compound is a member of a novel class of recently developed membrane-impermeant derivatives which selectively inhibit membrane-bound isozyme CA IV. [6]

Recently, our groups proved that metal complexes of heterocyclic sulfonamides behave as even stronger CA inhibitors,[7,8] and their mechanism of action was also explained, as being due to a dual inhibition by sulfonamido anions and metal ions, formed by dissociation of the complex inhibitor, which subsequently bind to different sites of the enzyme.[9] Metal ions probably bind to catalytically critical histidine residues[10,11] whereas the sulfonamido anions coordinate to the Zn(II) ion within CA active site.[12]

A large series of such complexes was reported in the last years, containing mainly acetazolamide **1**, methazolamide **2** and ethoxzolamide **3** as ligands, and structures were determined for some of them by means of X-ray crystallography and spectroscopic methods,[7,8,13,14] although some other ligands were investigated too.[1,7,14] As the coordination chemistry of this type of ligands is sometimes complicated due to their versatility and large number of donor atoms present in their molecules, it is of great interest to investigate some other ligands, as well as ternary complexes containing heterocyclic sulfonamides and amine-type ligands, also due to their possible pharmacological applications.[2,7]

In this paper we report the preparation and characterization by spectroscopic, magnetic, conductimetric and thermogravimetric methods of Ni(II) complexes of sulfonamides **1-8** and hydrazine as ligands. The prepared complexes behave as very efficient inhibitors of human isozymes CA I and CA II.

---

[#] Presented in part at the European Research Conference "Chemistry of Metals in Biological Systems", San Miniato, Italy, 1995.
*Correspondence author.

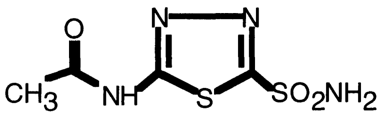

1: $H_2$aaz

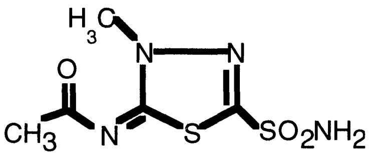

2: Hmza

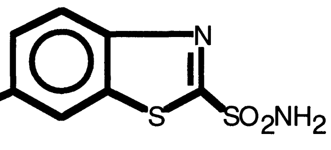

3: Heza

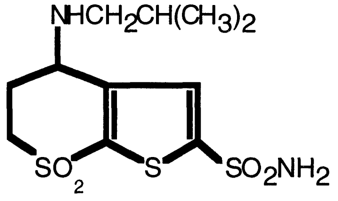

4: Hsza

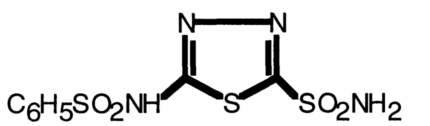

5: $H_2$bza

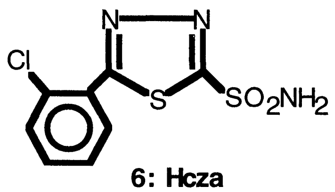

6: Hcza

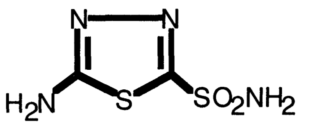

7: Htda

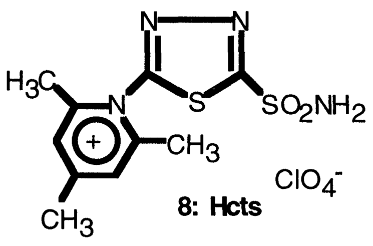

8: Hcts

### Materials and Methods

IR spectra were recorded on a Perkin Elmer 881 instrument, in the range 200-4000 $cm^{-1}$, in KBr pellets. Electronic spectra were obtained by the diffuse reflectance technique in MgO as reference, with a Perkin Elmer Lambda 15 apparatus, in the range 200-900 $cm^{-1}$. Conductimetric measurements were done in DMF solutions, at 25°C (concentrations of 1 mM of complex) with a Fisher conductimeter. Magnetic susceptibility measurements were carried out at room temperature with a fully automized AZTEC DSM8 pendulum-type susceptometer. Mercury(II) tetrakis(thiocyanato)cobaltate(II) was used as a susceptibility standard. Corrections for the diamagnetism were estimated from Pascal's constants.[15] Elemental analyses were done by combustion for C,H,N with an automated Carlo Erba analyzer, and gravimetrically for the metal ions, and were ± 0.4% of the theoretical values. Thermogravimetric measurements were done in air, at a heating rate of 10°C/min., with a Perkin Elmer 3600 thermobalance.

Acetazolamide, methazolamide and human CA I and CA II were from Sigma. Sulfonamides **3,4,6-8** used in the syntheses were prepared as described in the literature.[5,16] Metal salts, organic reagents used for preparing the sulfonamides and solvents were from Aldrich and were used without additional purification. Benzolamide **5** was a gift from Dr. T.H.Maren (University of Florida, Gainesville). Inhibitors were assayed by Maren's micromethod [17], in the conditions of the E-I (enzyme-inhibitor) technique, at 0°C in veronal buffer. $IC_{50}$ values represent the molarity of inhibitor producing a 50% decrease of CA specific activity for the $CO_2$ hydration reaction.

### Synthesis of coordination compounds 9-16

10 mMoles of sulfonamide **1-8** were dissolved in the minimum amount of ethanol (generally 50-200 mL).

To this solution was added 10 mL of aqueous solution containing 5 mMoles Ni(II) salt (nitrate, or chloride) and the obtained solution was treated with an excess (3 mL) of hydrazine hydrate 80%. The mixture was stirred magnetically at room temperature for 2 hours, then the obtained precipitates were filtered and air-dried. Yields were in the range of 56-95%.

### Results and Discussion

The prepared complexes and their proposed formulas (based on elemental analysis, 0.5% of the theoretical values) are shown in Table I.

Table I : Prepared complexes **9-16**, containing hydrazine and heterocyclic sulfonamides, and their elemental analysis data..

| No. | Complex | Color | Yield (%) | Analysis (calculated/found) %M[a] | %C[b] | %H[b] | %N[b] |
|---|---|---|---|---|---|---|---|
| **9** | $[Ni_2(aaz)_2(N_2H_4)_2]$ | violet | 56 | 18.8/18.5 | 15.4/15.1 | 3.5/3.3 | 26.9/26.7 |
| **10** | $[Ni_2(mza)_2(N_2H_4)_2(OH)_2]$ | violet | 45 | 16.9/17.1 | 17.5/17.4 | 3.5/3.3 | 24.5/24.5 |
| **11** | $[Ni(eza)(N_2H_4)_2(OH)]$ | lilac | 67 | 14.8/14.6 | 27.2/27.0 | 4.5/4.4 | 21.1/20.8 |
| **12** | $[Ni(sza)(N_2H_4)]$ | lilac | 42 | 14.1/14.1 | 28.8/28.5 | 5.0/5.2 | 13.4/13.0 |
| **13** | $[Ni(Hbza)(N_2H_4)_4(OH)]$ | violet | 57 | 11.1/10.7 | 18.4/18.0 | 4.6/4.6 | 32.1/31.9 |
| **14** | $[Ni(cza)(N_2H_4)(OH)]$ | violet | 75 | 15.1/15.2 | 25.1/25.1 | 2.6/2.6 | 18.3/18.1 |
| **15** | $[Ni(tda)(N_2H_4)_4(OH)]$ | lilac | 34 | 15.1/14.7 | 6.2/6.2 | 5.2/5.3 | 43.9/44.1 |
| **16** | $[Ni(cts)_2(N_2H_4)_2](ClO_4)_2$ | violet | 18 | 6.5/6.8 | 26.9/30.1 | 3.6/3.5 | 18.8/18.9 |

[a]By gravimetry; [b]By combustion.

The new compounds **9-16** were also characterized by means of spectroscopic (IR and electronic spectra), thermogravimetric, conductimetric and magnetic measurements. Some of these data are shown in Tables II and IV.

Table II: IR spectra, magnetic moments at room temperature, thermogravimetric and conductimetric data for complexes **9-16**.

| **Comp.** | **IR. Spectra**[a], $cm^{-1}$ $\Delta(SO_2)^s$ | $\Delta(SO_2)^{as}$ | $\mu_{eff}$[b], (BM) | **TG analysis**[c] found /calc. | **Conductimetry**[d] $\Lambda_M$ ($\Omega^{-1}$ x $cm^2$x $mol^{-1}$) |
|---|---|---|---|---|---|
| **9** | 5 | 14 | 2.34 | 10.2/10.1[e] | 12 |
| **10** | 10 | 12 | 2.56 | 9.2/9.3[e] | 10 |
| **11** | 10 | 15 | 2.89 | 16.0/16.1[e] | 9 |
| **12** | 7 | 18 | 3.43 | 7.7/7.5[f] | 17 |
| **13** | 21 | 20 | 2.40 | 24.7/24.5[g] | 8 |
| **14** | 9 | 17 | 2.91 | 8.3/8.3[f] | 12 |
| **15** | 8 | 14 | 3.68 | 33.3/33.5[g] | 15 |
| **16** | 12 | 19 | 4.10 | 7.2/7.0[e] | 285[h] |

[a] In KBr pellets; $\Delta(SO_2) = (SO_2)_{sulfonamide} - (SO_2)_{complex}$; [b] At room temperature; [c] Weight loss, % (only first step), corresponding to: [e]$2N_2H_4$; [f]$1N_2H_4$; [g]$4N_2H_4$ ; [d] Solution 1 mM (DMF) at 25°C; [h] 1:2 electrolyte.

In the IR spectra of the complex derivatives, the main change as compared to the uncomplexed sulfonamides was the shift of the $SO_2$ vibrations (in the range 1100-1180 $cm^{-1}$ for the antisymmetric vibrations, and 1300 - 1376 $cm^{-1}$ for the symmetric vibrations, respectively, in derivatives **1-8**)[2,5-7] to lower wavenumbers (with 5 - 20 $cm^{-1}$), as for previously reported such complexes.[7,8,13-15] The thiadiazole bands around 1500 - 1600 $cm^{-1}$ are also slightly shifted to lower wavenumbers in the spectra of the complexes as compared to the corresponding sulfonamide (data not shown), suggesting a possible interaction of Ni(II) with some of the endocyclic nitrogens of compounds **1-8** too. Moreover, the hydrazine vibration,[18] around 970 $cm^{-1}$ ,was also detected in the IR spectra of complexes **9-16**.

Table III: Electronic spectral data of complexes **9-16**.

| Complex | Band ($cm^{-1}$)[a] | Calculated Parameters |
|---|---|---|
| **9** | 17,825 ($\nu_3$)<br>11,111 ($\nu_2$)<br>7,045.5 ($\nu_1$) | 10Dq = 7,045 $cm^{-1}$<br>B = 521.84 $cm^{-1}$<br>$\beta$ = 0.54 |
| **10** | 18,587 ($\nu_3$)<br>11,450 ($\nu_2$)<br>7,150 ($\nu_1$)<br>27,050 (sh) | 10Dq = 7,150 $cm^{-1}$<br>B = 571.90 $cm^{-1}$<br>$\beta$ = 0.53 |
| **11** | 18,747 ($\nu_3$)<br>11,507 ($\nu_2$)<br>7,170 ($\nu_1$)<br>28,329 (sh) | 10Dq = 7,045 $cm^{-1}$<br>B = 581.80 $cm^{-1}$<br>$\beta$ = 0.54 |
| **13**[b] | 17,690 ($\nu_3$)<br>11,111 ($\nu_2$)<br>7,076 ($\nu_1$) | 10Dq = 7,076 $cm^{-1}$<br>B = 505.43 $cm^{-1}$<br>$\beta$ = 0.47 |
| **14** | 18,818 ($\nu_3$)<br>11,655 ($\nu_2$)<br>7,342 ($\nu_1$)<br>24,570 (sh) | 10Dq = 7,341 $cm^{-1}$<br>B = 564.68 $cm^{-1}$<br>$\beta$ = 0.52 |
| **15** | 18,491 ($\nu_3$)<br>11,450 ($\nu_2$)<br>7,150 ($\nu_1$)<br>28,329 (sh) | 10Dq = 7,150 $cm^{-1}$<br>B = 571.91 $cm^{-1}$<br>$\beta$ = 0.53 |
| **16** | 19,870 ($\nu_3$)<br>11,480 ($\nu_2$)<br>5,280 ($\nu_1$) | 10Dq = 6,206 $cm^{-1}$<br>B = 1,034.41 $cm^{-1}$<br>$\beta$ = 0.96 |

[a] Band of the $\nu_1$ transition was calculated

As seen from data of Tables II, the effective magnetic moments of complexes **11**, **12**, **14** and **15** suggest an octahedral environment of Ni(II), whereas for **16**, a tetrahedral geometry of the metal ion is assumed. The other three complexes (**9**, **10** and **13**) have magnetic moments lower than the only spin component (2.83 BM), suggesting that any magnetic coupling between the two Ni(II) ions appears for the dinuclear derivatives, **9** and **10**.[19]

Reflectance diffuse (RD) spectra of the newly synthesized complexes are shown in Table III, together with some calculated parameters.

As seen from data of Table III, in the reflectance spectra of complexes **9-15**, a band appears in the visible range (around 18,000 $cm^{-1}$) and another one in the near infrared region, around 11,000 $cm^{-1}$, suggesting an octahedral environment of the Ni(II) ion(s).[19] The spectrum of compound **16** on the other hand shows a broad band centered at approximately 19,870 $cm^{-1}$ and another one at 11,480 $cm^{-1}$ , which indicate a distorted tetrahedral geometry of Ni(II) in this complex.[19] Table IV also shows the assignment of the bands using the Lever tables.[20] The results of RD spectra are thus in agreement with those of magnetic measurements, suggesting octahedral Ni(II) ions in all but the last complex (**16**), which presumably contains tetrahedral Ni(II).

The donor system of the conjugated base of sulfonamides **1-7** is probably constituted by the anionic nitrogen of the sulfonamido moiety and the endocyclic nitrogens, excepting for sezolamide **4** where probably the endocyclic sulfur participates in coordination. The positively-charged sulfonamide **8** on the other hand seems to act as monodentate ligand when deprotonated at the sulfonamido moiety, by means of the sulfonamido nitrogen. Hydrazine probably acts as both mono., bidentate and bridging bidentate ligand in the prepared complexes. TG and conductimetric data (Table II) are in agreement with the above proposals. As no appropriate crystals for X-ray diffraction experiments could be obtained, the precise structure of the prepared complexes is not assigned for the moment.

Biological activity data with the new complexes (compared to that of the sulfonamide from which they derive) are presented in Tables IV and V.

Table IV: Biological activity data of sulfonamide CA inhibitors and complexes containing hydrazine and metal ions ($IC_{50}$ represents the molarity of inhibitor producing a 50% decrease of enzyme specific activity for the $CO_2$ hydration reaction, by Maren's micromethod[17]).

| Compound | $IC_{50}$ (nM) CA I | CA II |
|---|---|---|
| **1** | 200 | 7 |
| **2** | 10 | 9 |
| **3** | 1 | 0.6 |
| **4** | $>5.10^6$ | 2 |
| **5** | 2 | 1 |
| **6** | 1 | 1 |
| **7** | 280 | 30 |
| **8** | 165 | 14 |
| **9** | 105 | 5 |
| **10** | 1.8 | 4 |
| **11** | 0.4 | 0.2 |
| **12** | 800 | 1 |
| **13** | 0.9 | 0.8 |
| **14** | 0.7 | 0.5 |
| **15** | 150 | 20 |
| **16** | 80 | 8 |

As seen from data of Table IV, the new complexes prepared by us behave as strong inhibitors for both investigated CA isozymes, with potencies larger than those of the corresponding sulfonamides. Generally, complexes were 1.1 - 5.5 times more active than the free sulfonamides from which they were prepared. The notable exception is **12**, which is more than 6000 times more inhibitory towards CA I, as compared to sezolamide **4**. No explanation for this large difference of activity is available for the moment, but in Table V some comparative data are shown regarding CA I inhibition with sezolamide **4**, Cu(II) ions as well as complex **12**. From such data, one can conclude that at least for isozyme CA I, the enzyme inhibition observed with this class of derivatives is due to the metal complex *per se*, and not to its dissociation products formed in dilute solution (metal ions and sulfonamido anions), as hypothesized eralier by us.[7,8]

Table V: CA I inhibition data with sezolamide **4**, Cu(II) ions (as copper(II) sulfate) and the complex **12**.

| System | %CA I activity [a] |
|---|---|
| CA I[b] + **4**[c] | 98 |
| CA I[b] + $Cu^{2+}$[d] | 91 |
| CA I[b] +( **4** + $Cu^{2+}$)[e] | 90 |
| CA I[b] + **12**[f] | 50 |

[a]CA activity in the same conditions and in the absence of inhibitors is taken as 100%.

[b] In all experiments [CA I] = 10 nM, 50 mM veronal buffer (pH 7.9).

[c] [**4**] = 0.8 μM

[d] [$Cu^{2+}$] = 0.8 μM

[e] [**4**] = [$Cu^{2+}$] = 0.8 μM

[f] [**12**] = 0.8 μM.

The increased activity as enzyme inhibitors of metal complexes of sulfonamides might be thus accounted on their direct binding to the enzyme, in undissociated formed, in addition to the previous explanation that the effect is due to a dual inhibition by metal ions and sulfonamido anions formed in dilute solution by dissociation of the complex.[7,8]

### Acknowledgements

The financial support of the supplementary agreement No. CIPDCT 940051 to the contract No. CHRXCT 920072 (MASIMO network) of CEE is gratefully acknowledged.

### References

1. Part 5 of this series: J. Borràs, T.Cristea and C.T.Supuran, *Main Group Met.Chem., in press.*
2. a)C.T.Supuran, "Carbonic anhydrase inhibitors", in "Carbonic Anhydrase and Modulation of Physiologic and Pathologic Processes in the Organism", I.Puscas Ed., Helicon, Timisoara, 1994, pp. 29-113; b) T.H.Maren, B.W.Clare and C.T.Supuran, *Roum. Chem. Quart. Rev.*, **1994**, *2*, 259-282.
3. T.H.Maren, in "Orphan Drugs", F.E..Karch Ed., M.Dekker, New York, 1982, pp. 89-115.
4. T.H.Maren, *Physiol.Rev.*, **1967**, *47*, 595-782.
5. J.J.Baldwin, G.S.Ponticello, P.S.Anderson, M.E.Christy, M.A.Murcko, W.C.Randall, H.Schwamm, M.F. Sugrue,J.P. Springer, P. Gautheron, J. Grove, P. Mallorga, M.P.Viader, B.M.McKeever, and M.A. Navia, *J.Med.Chem.*, **1989**, *32*, 2510-2513.
6. C.T.Supuran, G.Manole, A.Dinculescu, A.Schiketanz, M.D.Gheorghiu, I.Puscas and A.T.Balaban, *J. Pharm. Sci.*, **1992**, *81*, 716-719; b) C.T.Supuran and B.W.Clare, *Eur. J. Med. Chem.*, **1995**, *30*, 687-696.
7. G.Alzuet, S.Ferrer, J.Borràs and C.T.Supuran, *Roum.Chem.Quart.Rev.*, **1994**, *2*, 283-300.
8.a) C.T.Supuran, *Rev.Roum.Chim.*, **1992**, *37*, 849-855; b) C.T.Supuran, G.Manole and M.Andruh, *J.Inorg.Biochem.*, **1993**, *49*, 97-103.
9.a) S.Ferrer, G.Alzuet and J. Borràs, *J.Inorg.Biochem.*, **1989**, *37*, 163-174; b) C.Luca, M. Bărboiu and C.T.Supuran, *Rev.Roum.Chim.*, **1991**, *36*, 1169-1173.
10. D.N.Silverman and S.Lindskog, *Acc.Chem.Res.*, **1988**, *21*, 30-36.
11. C.T.Supuran, *Main Group Metal Chem., in press.*
12. A.E.Eriksson and A.Liljas, in "The Carbonic Anhydrases", S.J.Dodgson et al. Eds., Plenum, New York, **1991**, pp. 33-48.
13. a)U.Hartmann and H.Vahrenkamp, *Inorg.Chem.*, **1991**, *30*, 4676; b) S.Ferrer, J.G.Haasnoot, R.A.G. de Graaff, J.Reedijk and J.Borràs, *Inorg.Chim.Acta*, **1992**, *192*, 129-138; c) G.Alzuet, L.Casella, A. Perotti and J.Borràs, *J.Chem.Soc.Dalton Trans.*, **1994**, 2347-2351; d) G.Alzuet, J.Casanova, J.A.Ramirez, J.Borràs and O.Carugo, *J.Inorg.Biochem.*, **1995**, *57*, 219-234.
14.a) A.Antoniu, C.T.Supuran, and M.Brezeanu, *Rev.Roum.Chim.*, **1995**, *40*, 203-207; b) L. Sumalan, J.Casanova, G.Alzuet, J.Borràs, A.Castineiras and C.T.Supuran, *J.Inorg.Biochem., in press;* c) C.T.Supuran, *Metal Based Drugs*, **1995**, *2*, 327-330; d) C.T.Supuran, *Metal Based Drugs*, **1995**, *2*, 331-336; e) C.T.Supuran, *Metal Based Drugs*, **1996**, *3*, 25-30.
15. R.S.Drago, in "Physical Methods in Chemistry", W.B.Saunders & Co., London, 1977, p. 411.
16. a) J.Korman, *J.Org.Chem.*, **1958**, *23*, 1768-1772; b) R.O.Roblin, and J.W.Clapp, *J.Am.Chem.Soc.*, **1950**, *72*, 4890-4892; c) C.T.Supuran, M.A.Ilies, T.B.Tewson and E.R.Swenson, *J.Med.Chem.*, in press.
17. T.H.Maren, *J.Pharmacol.Exp.Ther.*, **1960**, *130*, 26 -30.
18. A.K.Srivastava, A.L.Varshney and P.C.Jain, *J.Inorg.Nucl.Chem.*, **1980**, *42*, 47-50.
19. L.Sacconi, F.Mani and A.Bencini, "Nickel", in "Comprehensive Coordination Chemistry", G.Wilkinson, R.Gillard and J.McCleverty Eds., Pergamon Press, Oxford, 1987, Vol. 5, pp. 1-347.
20. A.B.P.Lever, "Inorganic Electronic Spectroscopy", 2nd edition, Elsevier, Amsterdam, 1984.

**Received: April 19, 1996 - Accepted: April 28, 1996 - Received in revised camera-ready format: April 30, 1996**

